# Association between the systemic immune-inflammation index and sarcopenia: a systematic review and meta-analysis

**DOI:** 10.1186/s13018-024-04808-7

**Published:** 2024-05-28

**Authors:** Siye Xie, Qi Wu

**Affiliations:** 1 School of Nursing, Zhejiang Chinese Medical University, Hangzhou, 310000 China; 2 Department of Nursing, The First Affiliated Hospital, Zhejiang University School of Medicine, No. 79 Qingchun Road, Hangzhou, Zhejiang 310003 China

**Keywords:** Systemic immune-inflammation index, Sarcopenia, Meta-analysis, Systematic review

## Abstract

**Background:**

Sarcopenia is associated with increased morbidity and mortality. The systemic immune-inflammation index (SII) has been correlated to a variety of disorders. The present study conducted a systematic review and meta-analysis to investigate the relationship between SII and sarcopenia.

**Methods:**

A literature search was performed in Web of Science, PubMed, Embase, Cochrane Library, CINAHL, China National Knowledge Infrastructure, Chinese Biomedical Literature Database, Wanfang Database, and VIP Chinese Science and Technology Database, from inception to March 2024. Then, the literature quality was assessed. After the heterogeneity test, a random effects or fixed effects model was applied to establish the forest plot, and investigate the relationship between SII and sarcopenia. Then, the sensitivity analysis and publication bias were examined.

**Results:**

Nine articles, which included 18,634 adults, were analyzed. Sarcopenic adults had higher SII levels, when compared to non-sarcopenic adults (standardized mean difference [SMD] = 0.66, 95% confidence interval [CI] = 0.22 − 0.19, *p* = 0.003). The high SII level was associated to the increased risk of sarcopenia (odds ratio = 1.52, 95% CI = 1.09–2.13, *p* = 0.01). In addition, the subgroup analysis revealed that the SII levels were higher in the sarcopenic group, when compared to the non-sarcopenic group, in elderly adults, as well as in adults with or without gastrointestinal disorders. The analysis was robust with a low risk of publication bias.

**Conclusions:**

SII is closely associated to sarcopenia. Sarcopenic adults had elevated SII levels. The high SII level increased the risk of sarcopenia. Large scale multi-center prospective studies are required to validate these study findings.

## Background

Sarcopenia refers to a syndrome characterized by the age-related progressive and systemic loss of skeletal muscular mass and strength [1–3]. Affected individuals present with functional decreases, and increased risk for falls, fractures, physical limitations, low quality of life, and even death [4]. Depending on the definition used and population tested, approximately 10–27% of people can have sarcopenia worldwide [5]. Sarcopenia is more frequently detected in elderly adults, and is associated to a variety of illnesses, such as metabolic disorders, cardiovascular disease, diabetes, and dementia [6–10]. The early identification and management of sarcopenia may prevent related comorbidities [11]. The underlying pathogenesis of sarcopenia remains unknown. Since dysregulated inflammatory response has been frequently reported to be associated to the reduction in muscle mass and physical function in adults with sarcopenia, inflammatory biomarkers may be used to understand the pathogenesis of sarcopenia, and provide early diagnosis and prognosis prediction [12–14]. For example, a number of pro-inflammatory cytokines have been proposed to decrease muscle mass by increasing muscle protein breakdown, and reduce muscle protein synthesis, leading to sarcopenia [15, 16]. However, there is no consensus on a single cytokine biomarker or pathway that could be responsible for the development of sarcopenia.

The systemic immune-inflammation index (SII) is a promising noninvasive multi-marker index calculated based on the blood cell count for neutrophils, lymphocytes and platelets [17]. Compared to a single inflammatory cytokine, SII has better sensitivity and specificity in evaluating the host overall inflammatory and immune extent and severity. Furthermore, this is a noninvasive, inexpensive, easy to calculate, and widely available biomarker. SII is associated to the prognosis of various types of malignancies, chronic obstructive pulmonary disease, and hepatic steatosis [18–21]. Furthermore, recent studies have reported SII as a promising biomarker for the development of cardiovascular diseases, stroke, psoriasis, insulin resistance, and ulcerative colitis [22–26]. Several cohort and cross-sectional studies have investigated the relationship between SII and sarcopenia. However, there has been no consensus on the study results.

Therefore, the present study conducted a meta-analysis, and systematically reviewed published studies that investigated the relationship between SII and sarcopenia. The present study aims to elucidate the association between SII and sarcopenia, and investigate SII as a biomarker for the prediction and diagnosis of sarcopenia, and for early therapeutic interventions and maintenance of quality of life.

## Methods

### Literature search and eligibility criteria

A systematic literature search was performed for the following databases that published studies in the Chinese and English languages, from inception to March 2024: Web of Science, PubMed, Embase, Cochrane Library, CINAHL, China National Knowledge Infrastructure, Chinese Biomedical Literature Database, Wanfang Database, and VIP Chinese Science and Technology Database. The following keywords, combined with the Medical Subject Headings terms and free words, were used: “systemic immune-inflammation index”, “SII”, and “sarcopenia/sarcopeni*/muscle wast*/muscle mass/muscle loss/muscle weakness/muscular atrophy”. The Chinese keywords used for the search were the Chinese translation of the above words. The meta-analysis was registered in PROSPERO (CRD42024518530).

Literature inclusion criteria: (1) human studies with a participant age of > 18 years old; (2) studies with an observational study design; (3) studies that investigated the relationship of SII and sarcopenia; (4) studies that reported the outcome evaluations for SII measurements between the sarcopenia group and non-sarcopenia group, or the incidence of sarcopenia between the high- and low-SII groups. Literature exclusion criteria: (1) studies that included systemic inflammation-related diseases and inflammatory drug therapies; (2) duplicate publications; (3) the research data of the study was not accessible or not available; (4) reviews, case studies, conference abstracts, and articles without the full text.

### Literature selection and data extraction

Two researchers independently exported the articles to EndNote (version X9; Clarivate, USA), according to the eligibility criteria. Any disagreements between the two researchers were resolved by discussion with a third researcher to reach a final consensus. The extracted data included the following: name of the first author, publication year, country, study design, participant characteristics, sample size, age, sarcopenia diagnostic criteria, comorbidities, and outcome measurements.

### Literature quality assessment

Two researchers assessed the quality of the included literature based on the Newcastle-Ottawa Quality Scale (NOS) and Joanna Briggs Institute (JBI) Evidence-Based Healthcare quality assessment tool for cross-sectional studies [27, 28]. Any disagreements were discussed with a third researcher. The NOS included eight items in three domains, with a maximum score of 9. Studies with scores of 0–5 and 6–9 were considered low-quality and high-quality studies, respectively. The JBI Evidence-Based Healthcare quality evaluation tool for cross-sectional studies included eight items to assess the overall quality of a study.

### Statistical analysis

All statistical analyses were performed using RevMan 5.4 (Cochrane, USA). The inter-group differences were the standardized mean difference (SMD) for continuous variables, or the odds ratio (OR) with 95% confidence interval (CI) for categorical variables. Chi-square test and *I*^*2*^ statistic were used to assess the heterogeneity of the included studies. When *p* > 0.1 and *I*^*2*^ < 50%, the heterogeneity was considered small, and a fixed effects model was selected for the analysis. When *p* ≤ 0.1 and *I*^*2*^ ≥ 50%, the heterogeneity was considered significant, and the source of heterogeneity was further determined. After sequentially excluding the article that caused the potential clinical heterogeneity, a random effects model was applied for the analysis. A subgroup analysis was conducted for studies with different sarcopenia definitions and age groups, and studies with or without gastrointestinal disorders. Finally, the sensitivity analysis and publication bias were examined.

## Results

### Characteristics of the included literature

**Fig. 1:**
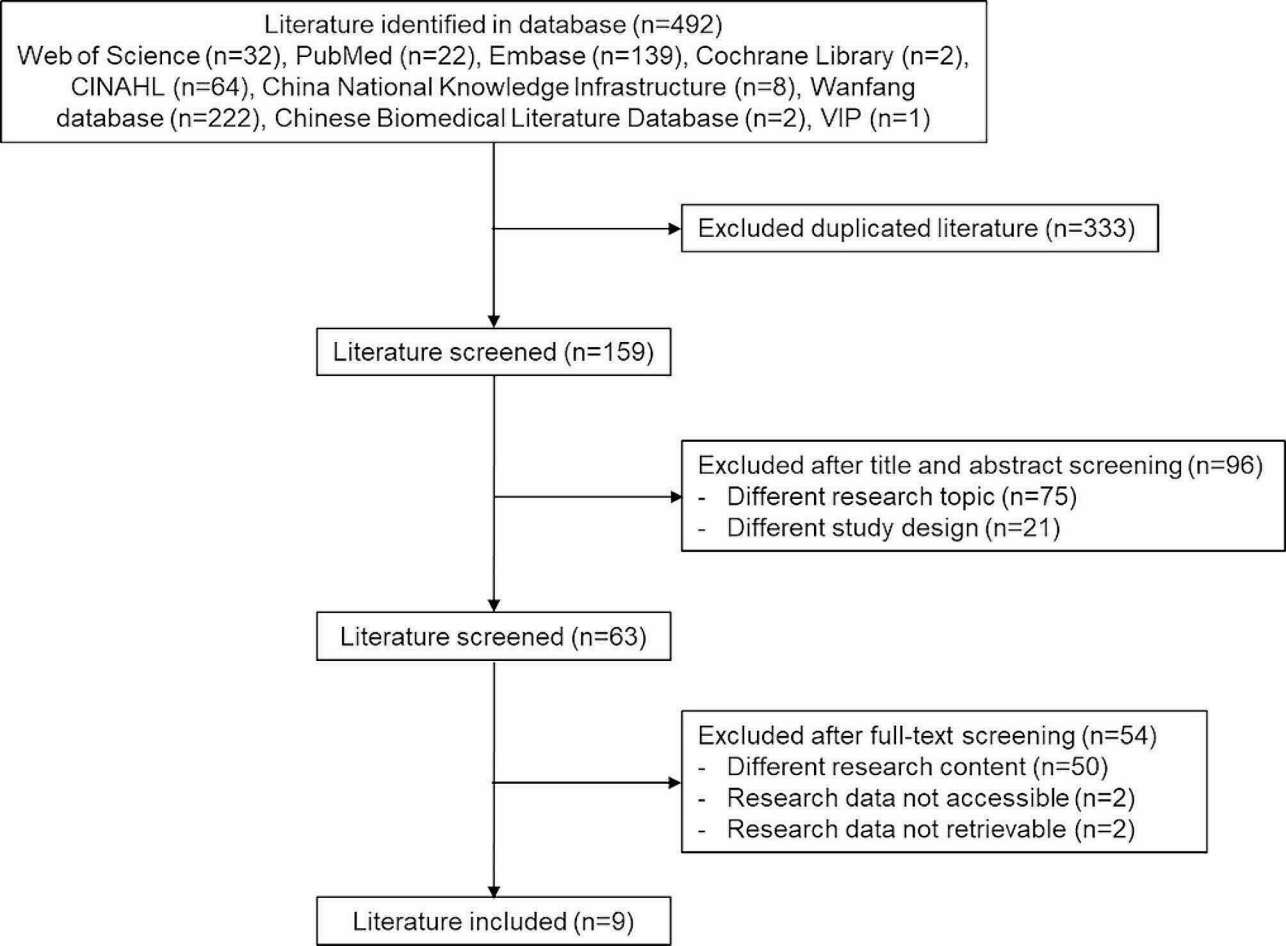
Flowchart for the literature selection

A total of 492 studies were identified by the initial literature search, and nine studies were included for the final analysis (Fig. 1). These nine studies included 18,634 participants (Table 1).

**Table 1:** Characteristics of the included literature

Author	Year	Country	Study design	Participants	Age (study / control group)	Sample size (study / control group)	Sarcopenia definition
Jiang et al. [29]	2024	China	Cohort	Elderly	75.5 ± 7.3 / 70.9 ± 5.2	377 (66 / 311)	A
Wu et al. [30]	2023	China	Cohort	Inflammatory bowel disease	32.0 (21.5, 49.5) / 46.0 (33.0, 57.0)	108 (81 / 27)	B
Chen et al. [31]	2023	China	Cross-sectional	Colon cancer	67 (57, 73) / 59 (51, 69)	410 (232 / 178)	B
Shi et al. [32]	2023	USA	Cross-sectional	Adults	39.4 ± 0.3	10,367 (5,184 / 5,183)	C
Ding et al. [33]	2022	China	Cohort	Locally advanced gastric cancer	58.4 ± 8.7	134 (31 / 103)	A
Zhao et al. [34].	2021	China	Cohort	Middle-age and elderly	62.3 ± 8.2	4,224 (814 / 3,410)	A
Karanth et al. [35]	2021	USA	Cross-sectional	Chronic illness	70.2 + 7.5	2,483 (1,243 / 1,240)	D
Li et al. [36]	2021	China	Cohort	Under chemoradiation therapy after radical gastrectomy	54 (49, 62)	223 (138 / 85)	E
Okugawa et al. [37]	2019	Japan	Cohort	Colorectal cancer	≤ 67, 159; >67, 149	308 (224 / 84)	F

The diagnosis of sarcopenia was based on the following: (A) the 2019 criteria of AWGS [38], including (1) muscle strength estimated using handgrip strength, with cut-off values of 28 kg for men and 18 kg for women; (2) the skeletal muscle index (SMI) determined by bioelectrical impedance analysis, with cut-off values of 7.0 kg/m^2^ for men and 5.7 kg/m^2^ for women (sarcopenia was diagnosed when the subject had reduced muscle mass and reduced muscle strength); (B) the SMI at the third lumbar level, with SMI < 52.4 cm^2^/m^2^ for men and SMI < 38.5 cm^2^/m^2^ for women to consider sarcopenia; (C) the appendicular skeletal muscle index, with < 0.789 m^2^ for men and < 0.512 m^2^ women to consider sarcopenia; (D) the European Working Group on Sarcopenia in Older People [39], with appendicular lean mass/height^2^ < 7.26 kg/m^2^ in men and appendicular lean mass/height^2^ < 5.45 kg/m^2^ in women to consider sarcopenia; (E) the SMI at the third lumbar level, with SMI < 37.6 cm^2^/m^2^ for men and SMI < 30 cm^2^/m^2^ for women to consider sarcopenia; (F) the psoas muscle mass index (PMI), with PMI < 286.3 m^2^ for men and PMI < 210.6 m^2^ for women to consider sarcopenia

### Quality assessment of the included literature

Among the nine studies, six cohort studies received a NOS score of 8, and three cross-sectional studies received “yes” on the JBI Evidence-Based Healthcare quality evaluation tool for cross-sectional studies (Tables 2 and 3).

**Table 2:** Evaluation of the included cohort literature using the Newcastle-Ottawa Quality Scale

Author	Selection				Comparability	Outcome			Total score
	Representativeness of exposed	Representativeness of unexposed	Ascertainment of exposure	Outcome not present at start of study		Blinded independent evaluation	Adequate follow-up period	Adequacy of follow-ups	
Jiang et al. [29]	1	1	1	1	2	0	1	1	8
Wu et al. [30]	1	1	1	1	2	0	1	1	8
Ding et al. [33]	1	1	1	1	2	0	1	1	8
Zhao et al. [34]	1	1	1	1	2	0	1	1	8
Li et al. [36]	1	1	1	1	2	0	1	1	8
Okugawa et al. [37]	1	1	1	1	2	0	1	1	8

**Table 3:** Evaluation of the included cross-sectional literature by Joanna Briggs Institute Evidence-Based Healthcare quality assessment

Author	A	B	C	D	E	F	G	H
Chen et al. [31]	Yes	Yes	Yes	Yes	Yes	Yes	Yes	Yes
Shi et al. [32]	Yes	Yes	Yes	Yes	Yes	Yes	Yes	Yes
Karanth et al. [35]	Yes	Yes	Yes	Yes	Yes	Yes	Yes	Yes

(A) Determination of whether the inclusion criteria for the research subjects were clearly defined; (B) Determination of whether the research subjects and research sites were described in detail; (C) Determination of whether standard, effective and credible methods were used to measure the exposure factors; (D) Determination of whether objective and standard methods were used to measure health issues; (E) Determination of whether the confounding factors were clarified; (F) Determination of whether measures were taken to control the confounding factors; (G) Determination of whether effective and credible methods were used to evaluate the outcome indicators; (H) Determination of whether the data analysis method was appropriate

### The relationship between Sarcopenia and SII

#### SII levels in adults with or without Sarcopenia

**Fig. 2:**
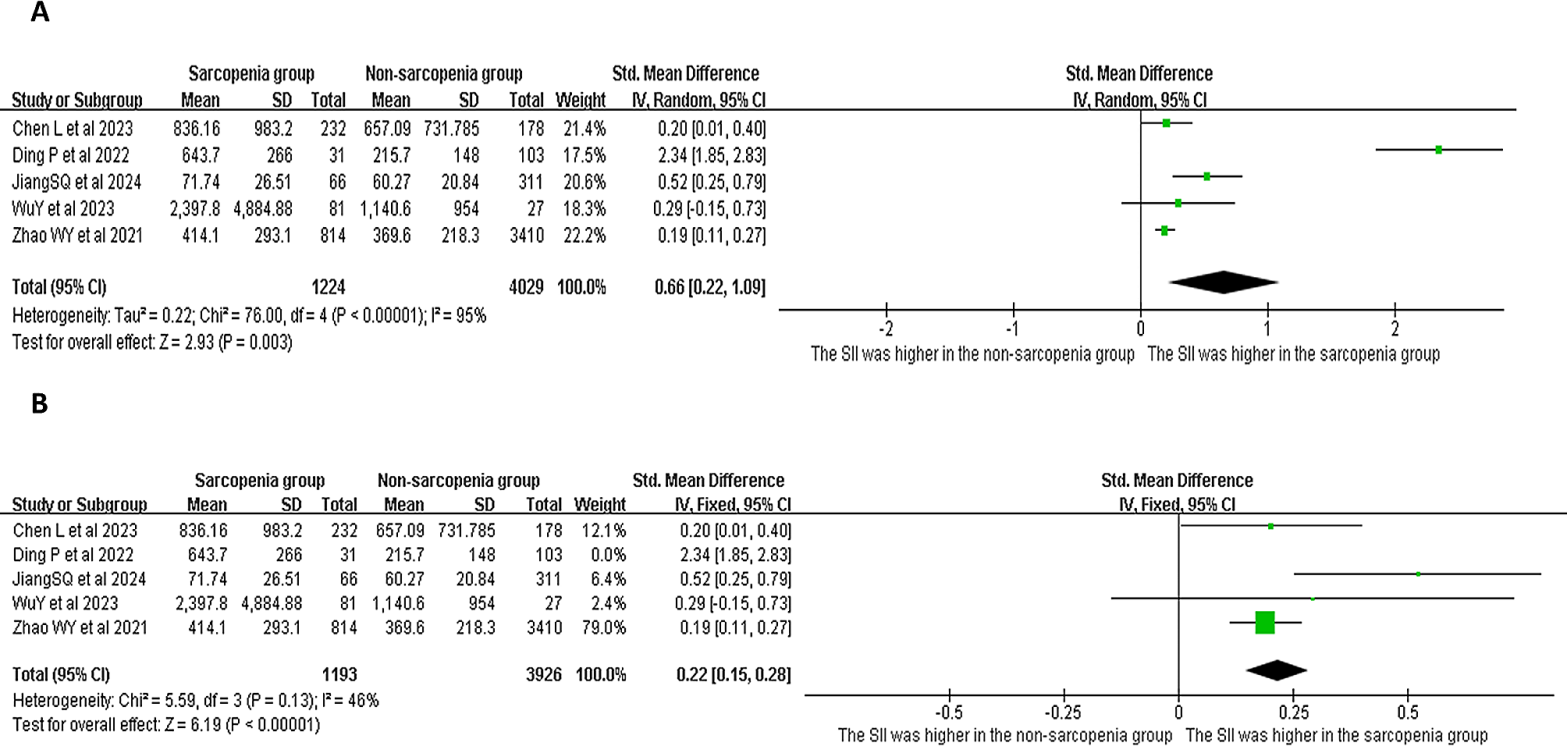
The forest plots show the SII levels for adults with and without sarcopenia (**A**: all five studies; **B**: after removing the study conducted by Ding et al.)

Five studies compared the SII measurements between adults with or without sarcopenia (Fig. 2). A random effects model was applied for the analysis due to high heterogeneity (*p* < 0.001, *I*^*2*^ = 95%). The results revealed that the SII score was higher in the sarcopenia group, when compared to the non-sarcopenia group (SMD = 0.66, 95% CI = 0.22 − 0.19, *p* = 0.003; Fig. 2A). The heterogeneity decreased (*p* = 0.13, *I*^*2*^ = 46%) after removing the study conducted by Ding et al. However, the fixed effects model continued to show that the SII score was higher in the sarcopenia group, when compared to the non-sarcopenia group (SMD = 0.22, 95% CI = 0.15–0.28, *p* < 0.001; Fig. 2B).

#### Risk of Sarcopenia in adults with high or low SII levels

**Fig. 3:**
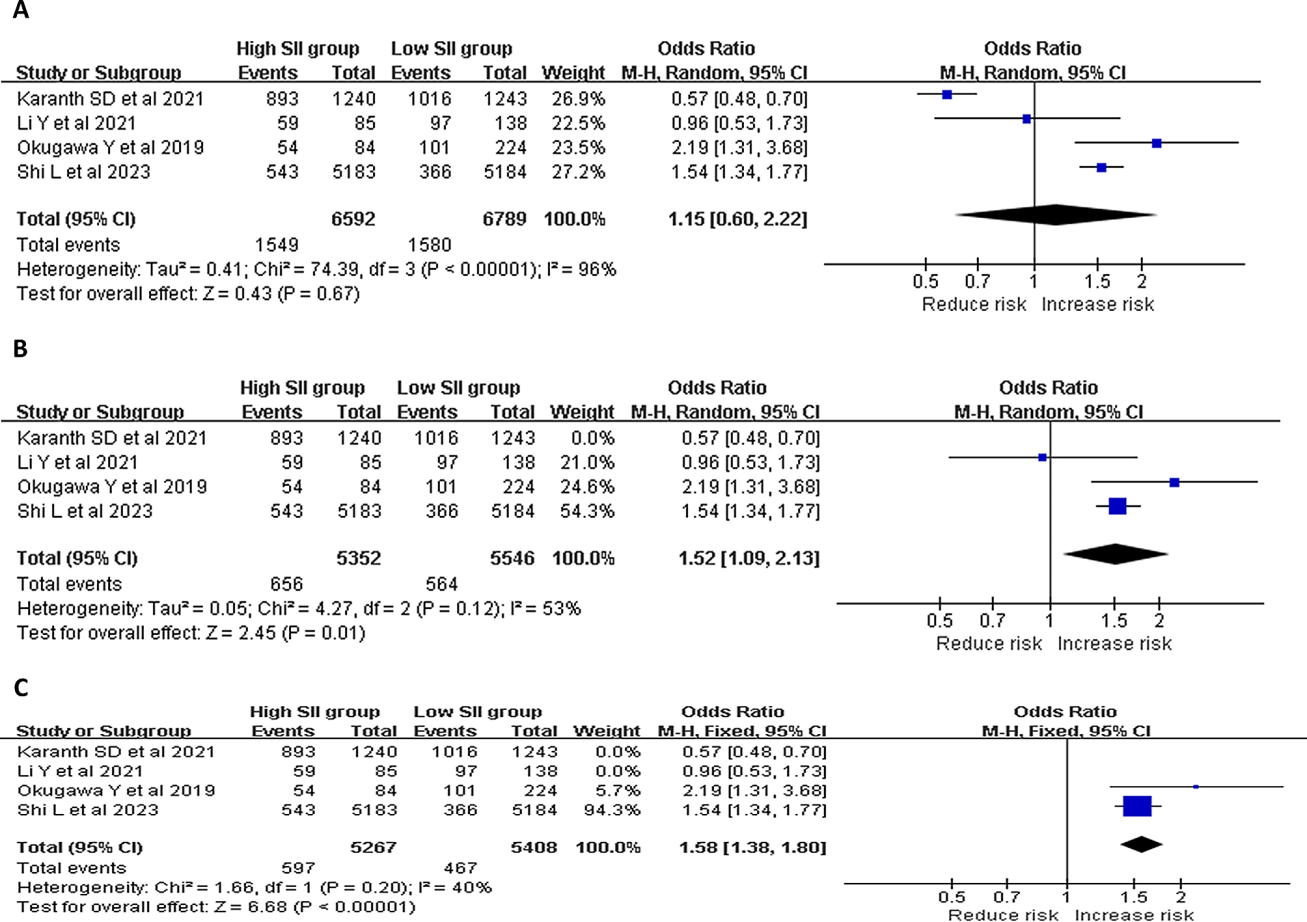
The forest plots show the risk of sarcopenia in adults with high and low SII levels (**A**: all four studies; **B**: after removing the study conducted by Karanth et al.; **C**: after removing the studies conducted by Karanth et al. and Li et al.)

Four studies compared the risk of sarcopenia between adults with high and low SII levels (Fig. 3). A random effects model was applied due to high heterogeneity (*p* < 0.001, *I*^*2*^ = 96%). There was no difference in the risk of sarcopenia between the high and low SII groups (OR = 1.15, 95% CI = 0.60–2.22, *p* = 0.67; Fig. 3A). A random effects model was applied again after removing the study conducted by Karanth et al. (*p* = 0.12, *I*^*2*^ = 53%). This revealed a statistically and significantly higher risk of sarcopenia in adults with high SII levels, when compared to those with low SII levels (OR = 1.52, 95% CI = 1.09–2.13, *p* = 0.01; Fig. 3B). The heterogeneity further decreased after removing the studies conducted by Karanth et al. and Li et al. (*p* = 0.20, *I*^*2*^ = 40%). The fixed effects model revealed that there was a statistically and significantly higher risk of sarcopenia in the high SII group, when compared to the low SII group (OR = 1.58, 95% CI = 1.38 = 1.80, *p* < 0.001; Fig. 3C).

### Subgroup analysis

#### Definition of Sarcopenia

**Fig. 4:**
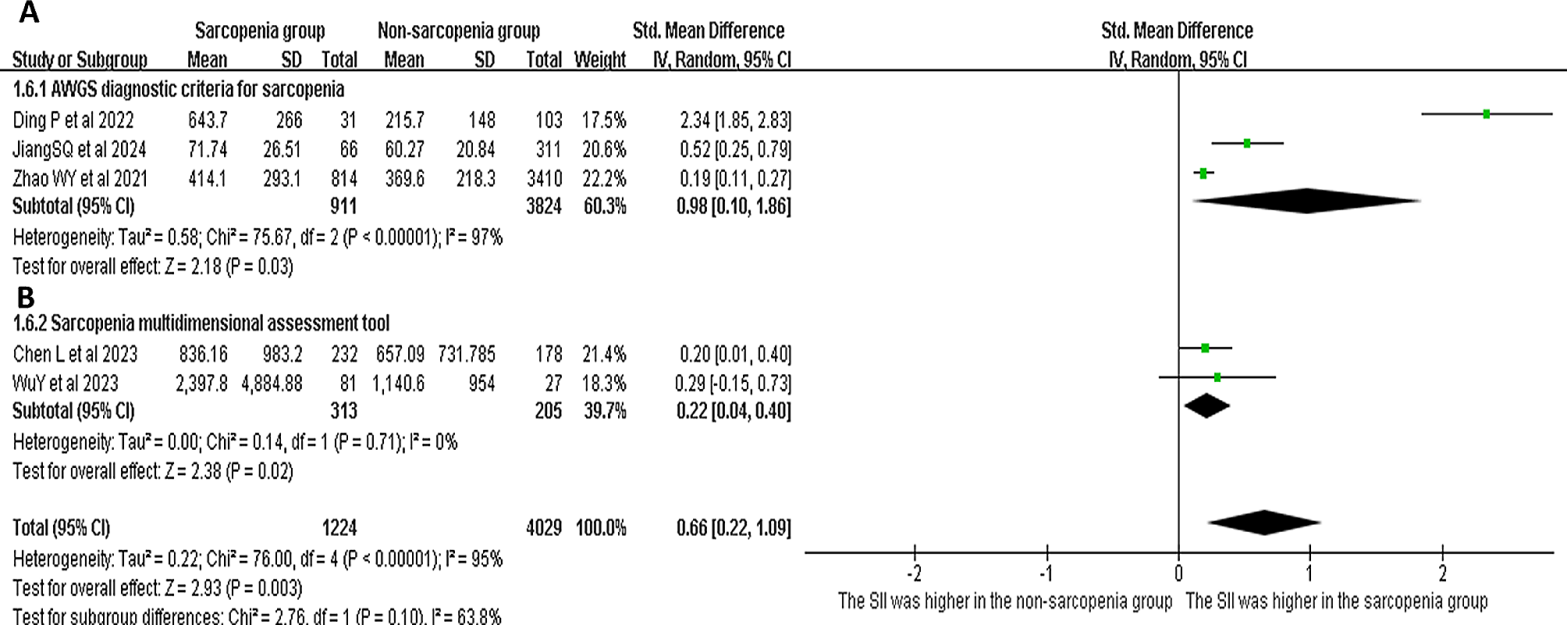
The forest plots show the SII levels in adults with and without sarcopenia (**A**: sarcopenia by AWGS definition; **B**: sarcopenia by SARC-F definition)

Among the five studies that compared the SII measurements between adults with or without sarcopenia, three studies used the Asian Working Group for Sarcopenia (AWGS), and two studies used the skeletal mass index at the third lumbar level to define sarcopenia (Fig. 4). A meta-analysis was separately performed for these two groups of studies with different definition criteria for sarcopenia. Both analysis results revealed higher SII levels in adults with sarcopenia, when compared to those without sarcopenia (*I*^*2*^ = 97%, SMD = 0.98, 95% CI = 0.10–1.86, *p* = 0.03; *I*^*2*^ = 0%, SMD = 0.22, 95% CI = 0.04–0.40, *p* = 0.02). A subgroup analysis was not performed for studies that compared the risk of sarcopenia between adults with high and low SII levels, since these studies used different definitions to diagnose sarcopenia.

#### Elderly population

**Fig. 5:**
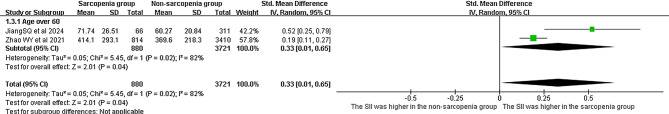
The forest plots show the SII levels in elderly adults with and without sarcopenia

Among the five studies that compared the SII measurements between adults with and without sarcopenia, two studies focused on the elderly population (age > 60 years old, Fig. 5). A random effects model was used due to high heterogeneity (*p* = 0.02, *I*^*2*^ = 92%). This revealed a high SII level in the sarcopenic group, when compared to the non-sarcopenic group (SMD = 0.33, 95% CI = 0.01–0.65, *p* = 0.04). Merely one study compared the risk of sarcopenia with the different SII levels in elderly adults. This study assigned these adults into four groups based on the following SII levels: <365.7, 365.7-503.6, 503.7–704.0, and > 704.1. The sarcopenia prevalence was 19.0%, 17.5%, 25.2% and 30.8%, respectively. Compared to the SII < 365.7 group, the risk of sarcopenia decreased in the SII 365.7-503.6 group (OR = 0.90, 95% CI = 0.68–1.20, *p* < 0.001), while the risk of sarcopenia increased in the SII 503.7–704.0 and SII > 704.1 groups (OR = 1.43, 95% CI = 1.09–1.88, *p* < 0.001; OR = 1.89, 95% CI = 1.45–2.46, *p* < 0.001).

#### Gastrointestinal disorders

**Fig. 6:**
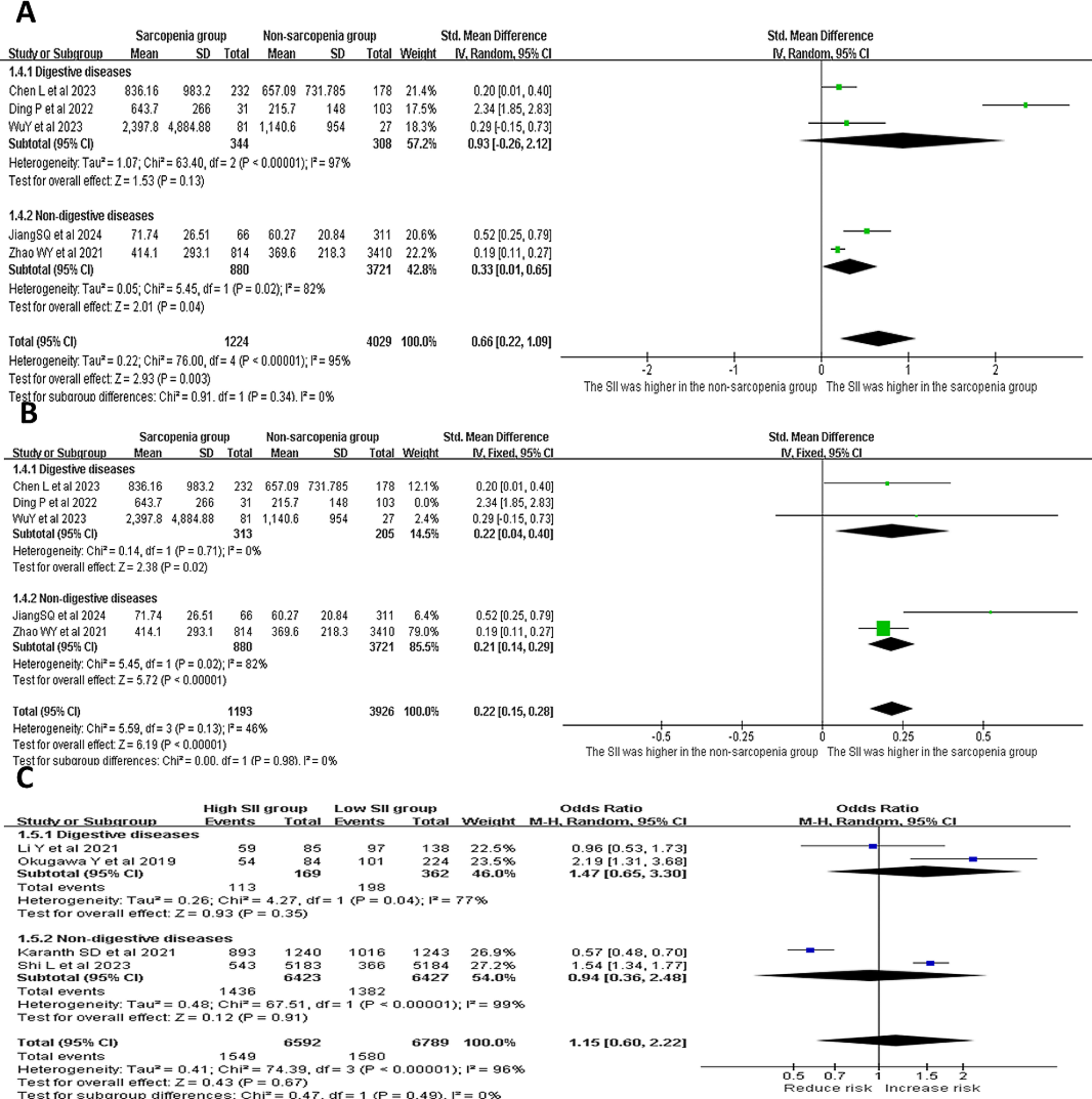
(**A** and **B**) The forest plots show the SII levels for adults with and without sarcopenia (**A**: all three studies; **B**: after removing the study conducted by Ding et al.). (**C**) The forest plots show the risk of sarcopenia in adults with high and low SII levels, with and without gastrointestinal disorders

Since gastrointestinal disorders can affect the nutrition supply, and is associated to the development of sarcopenia, a subgroup analysis was performed for adults with and without gastrointestinal disorders. Among the five studies that compared the SII measurements between adults with and without sarcopenia, three studies investigated adults with gastrointestinal disorders (Fig. 6). A random effects model was used due to high heterogeneity (*p* < 0.001, *I*^*2*^ = 95%). This revealed that there was no significant difference in SII measurements between adults with and without sarcopenia (SMD = 0.93, 95% CI = -0.26 to 2.12, *p* = 0.13; Fig. 6A). After removing the study conducted by Ding et al., the heterogeneity decreased (*p* = 0.71, *I*^*2*^ = 0%). The fixed effects model analysis revealed that the SII levels were higher in adults with sarcopenia, when compared to adults without sarcopenia, in both the gastrointestinal disorder group (SMD = 0.22, 95% CI = 0.04–0.40, *p* = 0.02) and non-gastrointestinal disorder group (*I*^*2*^ = 92%, SMD = 0.21, 95% CI = 0.14–0.29, *p* = 0.02) (Fig. 6B). In the subgroup analysis of studies that compared the risk of sarcopenia between adults with high and low SII levels, there was no association between the SII level and risk of sarcopenia in adults with and without gastrointestinal disorders (*I*^*2*^ = 77%, OR = 1.47, 95% CI = 065 − 3.30, *p* = 0.35; *I*^*2*^ = 99%, OR = 0.94, 95% CI = 0.36–2.48, *p* = 0.91; Fig. 6C).

#### Sensitivity analysis and publication bias

**Fig. 7:**
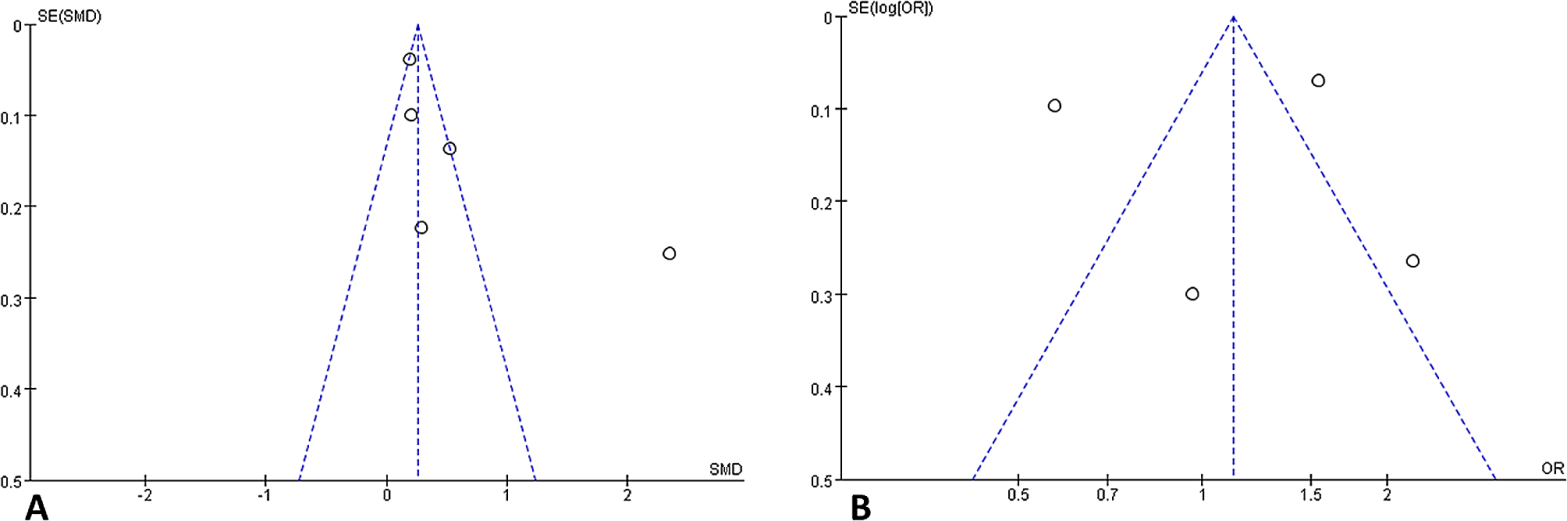
Funnel plot analysis. (**A**) Influence of sarcopenia on the SII level. (**B**) Influence of the SII level on the risk of sarcopenia

The robustness of the meta-analysis was confirmed by the no substantial change in results when an individual study was sequentially removed. Furthermore, the symmetrical funnel plot suggested a low risk of publication bias for the present analysis (Fig. 7).

## Discussion

Approximately one-fourth of the population can have sarcopenia [5]. Sarcopenia can decrease the quality of life, and increase the morbidity and mortality of affected adults. Early diagnosis and interventions can prevent its related comorbidities, and improve quality of life [40]. Chronic inflammation may play a role in the development of sarcopenia. The present meta-analysis revealed the close relationship between the SII level and sarcopenia. It was found that sarcopenic adults had elevated SII levels. A high SII level would increase the risk of sarcopenia. The further subgroup analysis confirmed this association in elderly adults, as well as in adults with and without gastrointestinal disorders.

Adults with sarcopenia have high SII levels. Sarcopenia can be categorized into primary and secondary sarcopenia [41]. Primary sarcopenia is age-related, and is commonly observed in adults of > 60 years old. These affected adults present with decreased muscle strength, mass and function, which occurs during the normal aging process. This is characterized by chronic systemic inflammation, with cellular senescence, immune impairment, and organ dysfunction [42]. Senescent cells can secrete the senescence-associated secretory phenotype to promote chronic inflammation, and induce normal cell senescence [43]. Chronic inflammation can, vice versa, accelerate the aging of immune cells. This would result in weakened immune function, and the inability to clear senescent cells and inflammatory factors, leading to a vicious cycle of inflammation and aging [44]. In the musculoskeletal system, this process can result in sarcopenia. The causes of secondary sarcopenia are diverse, and these include a variety of diseases, such as infections, injuries, surgeries and drugs [45]. Affected adults can present with a persistent process of muscle damage and repair, resulting in increased chronic inflammatory reactions in the body. Furthermore, adults with sarcopenia often present with malnutrition. The latter can lead to an increased risk of inflammatory reactions, with elevated oxidative stress and the release of reactive oxygen free radicals [46]. All these can lead to muscle damage and functional impairment, as well as illnesses to other organ systems. The relationship between sarcopenia and inflammation is complex, and requires further studies. The inflammation reactions may be decreased to reduce the severity of sarcopenia and other comorbidities.

The present analysis revealed that compared to the low SII group, the high SII group had a 1.58-fold increased risk of sarcopenia, suggesting that high SII is a risk factor for sarcopenia. The advantage of SII over other inflammatory indicators (such as C-reactive protein and interleukin-6) is its comprehensive evaluation and predictive performance. By considering the proportion of platelets, neutrophils and lymphocytes, SII can more comprehensively reflect the inflammatory state and immune function of the body [17]. In addition, SII has satisfactory performance in assessing disease progression and predicting prognosis, contributing to its wide application in clinical practice [18–26]. The high SII level indicates that adults have a higher degree of systemic inflammatory reaction. Inflammation is the human body’s biological response to injury, infection, or other stimuli, in order to maintain homeostasis, remove harmful substances, and promote damage repair. Furthermore, inflammation can serve as a marker, and plays an important role in the occurrence and development of various diseases [47, 48]. Chronic inflammation might be a major contributor to the pathogenesis of sarcopenia [49]. Furthermore, chronic inflammation creates a persistent, long-term inflammatory reaction that can lead to increased decomposition, and decreased synthesis of muscle protein, with ultimate muscle loss and mass reduction [50, 51]. Tuttle et al. reported that C-reactive protein, inerleukin-6, and tumor necrotic factor-α levels are significantly and negatively correlated to grip strength and knee joint extension strength [52]. A higher C-reactive protein level is significantly and negatively correlated to muscle mass. The association between higher levels of circulating inflammatory markers, and lower muscle strength and mass would vary depending on the race and gender. Hispanics and Asians are more likely to have sarcopenia, when compared to non-Hispanic Whites, which is probably due to racial disparities in inflammation reactions among different populations [53]. More research is needed to clarify the mechanisms underlying the association between chronic inflammation and sarcopenia, in order to develop therapeutic strategies that could block or minimize the inflammatory process, and decrease the risk of sarcopenia.

In 2016, the World Health Organization listed sarcopenia as one of the formal disease diagnoses in the 10th version of the International Classification of Diseases [54]. Several diagnostic criteria have been proposed for sarcopenia [55]. The European diagnostic criteria for sarcopenia are focused on clinical, biological, and imaging tests, with special attention placed on muscle injury and functional assessment, emphasizing the biological characteristics of muscle diseases [1]. The Asian diagnostic criteria for sarcopenia are more focused on genetic and electrophysiological examinations, emphasizing on genetic factors and neuro-muscle conduction function, and focusing more attention to genetic muscle disease screening in the Asian population [38]. These diagnostic criteria have its advantages and limitations. In China, AWGS is the commonly used diagnostic criteria for sarcopenia [38]. However, some measurements, such as grip strength and calf circumference, cannot be easily obtained. Different diagnostic criteria might lead to variations in determining sarcopenia and management strategies. Considering the potential racial differences in the risk of sarcopenia, further studies are required to identify more appropriate and easily accessible methods to diagnose sarcopenia in the Chinese population.

The prevalence of sarcopenia increases with the advance of age [56]. In the subgroup analysis of the elderly population, > 60 year-old adults with sarcopenia had higher SII levels, when compared to those without sarcopenia. The SII difference in these elderly adults with and without sarcopenia (0.33) was larger than the SII difference in the analysis of all-age adults with and without sarcopenia (0.22). This might be consistent with the study reports on accelerated muscle mass loss in the elderly population with sarcopenia. From the age of 50, muscle loss is estimated to be approximately 0.8% per year, which accelerates to approximately 2% per year starting from the age of 60 [46]. Aging leads to the reduction in the number and size of muscle fibers, and the decrease in the rate of protein synthesis in muscle tissues, resulting in the loss of muscle mass, lower muscle strength and endurance, slower muscle contractions, and reduced response [57]. All of these would finally affect the balance and daily activities [58]. An active lifestyle, including moderate exercise, a balanced diet, and adequate protein intake, might help to delay the onset, and reduce the extent of muscle loss [59]. The management of sarcopenia should be an important part of healthcare, in order to improve the quality of life of the elderly population.

Dietary nutrition intake is associated to sarcopenia [60]. The present subgroup analysis for adults with and without gastrointestinal disorders revealed higher SII levels in adults with sarcopenia, when compared to those without sarcopenia, in both subgroups. The human intestinal microbiota consists of 10–100 trillion microorganisms. This complex ecosystem plays a vital role in intestinal immune and endocrine functions, energy homeostasis, nutritional status, and health maintenance [61]. Based on the microbiota-gut-muscle axis theory, the intestinal microbiota can affect muscle quality and function by regulating inflammatory response, oxidative stress, energy metabolism, and insulin sensitivity [62]. Liu et al. conducted a systematic review on intestinal flora and sarcopenia. That study revealed that changing the intestinal microbiota through bacterial consumption, fecal transplantation, and various supplements can directly affect the muscle phenotype [63]. Probiotics, prebiotics, short-chain fatty acids, and beneficial bacteria are the potential new treatments to improve muscle mass and physical performance. Lactobacilli and bifidobacteria might restore age-related muscle loss. The gut flora can be a new target for the prevention and treatment of sarcopenia. However, due to significant individual variations in gut microbial composition, the correlation and mechanism between the gut flora and sarcopenia still needs to be further explored, which may lead to the development of personalized gut flora management strategy for individuals with sarcopenia.

Chronic inflammation can dysregulate the immune system and damage tissue structure, which finally affects organ functions, and causes various disorders. The present study results suggested that sarcopenia and inflammation might interact with each other. Adults with sarcopenia had higher SII levels, when compared to those without sarcopenia. A high SII level can increase the risk for sarcopenia. SII can be used as a biomarker to screen for sarcopenia. Adults with high SII levels should undergo further examinations, in order to rule out sarcopenia. Meanwhile, adults diagnosed with sarcopenia would frequently present with high SII levels. Since high SII levels are associated to various other chronic illnesses, sarcopenic adults with high SII levels should receive further evaluations for chronic illnesses, such as metabolic disorders, cardiovascular disease, diabetes and dementia. Traditional management for sarcopenia focuses on resistance training and protein supplementation [64]. The elucidation of the relationship between sarcopenia and inflammation can help to develop anti-inflammatory strategies. This may not only correct the muscle loss, but also reduce the risk for other chronic illnesses. In addition, other factors, such as age and nutritional status, should be considered when evaluating the relationship between inflammation status and sarcopenia. Inflammatory status is a dynamic process that requires constant re-assessments. Future research can further focus on the impact of SII-based inflammatory conditions on sarcopenia risk prediction models, and construct a more complete sarcopenia risk prediction model.

The strength of the present study was the comprehensive evaluation of the relationship between SII and sarcopenia in a large collective sample of adults. This not only confirmed this relationship in the adults included in the study, but also examined this relationship in the subgroup of elderly adults, and adults with and without gastrointestinal disorders. The limitation of the present study was that merely articles published in the English or Chinese language from the US, China and Japan were included. People who live in other countries might have different socioeconomic environments and lifestyles, which can affect the body inflammation status and muscle mass. Furthermore, some of the included studies were cross-sectional studies that could not evaluate the causal relationship between SII and sarcopenia. Moreover, the total number of included studies was small. In addition, each study used different diagnostic criteria and outcome measurements, which resulted in significant heterogeneity, especially in the subgroup analysis. All of these can lead to biases in the present analysis.

## Conclusions

In conclusion, there was a significant association between systemic inflammation reactions and sarcopenia. Adults with sarcopenia can have a high-level inflammatory status, and a high inflammatory status would increase the risk of sarcopenia. Future research is required to further elucidate the underlying mechanism and causal relationship between systemic inflammation reactions and sarcopenia. Conducting more subgroup analyses would help to develop individualized preventive strategies and therapeutic treatments for adults with sarcopenia and chronic systemic inflammation status.

## Data Availability

No datasets were generated or analysed during the current study.

## Abbreviations

AWGS: Asian Working Group for Sarcopenia
SMD: Standardized mean difference
CI: Confidence interval
SII: Systemic immune-inflammation index
NOS: Newcastle-Ottawa Quality Scale
JBI: Joanna Briggs Institute
OR: Odds ratio
